# Clinical Significance of Circulating Tumor Cells in Peripheral Blood of Patients with Esophageal Squamous Cell Carcinoma

**DOI:** 10.1245/s10434-015-4392-8

**Published:** 2015-02-05

**Authors:** Daisuke Matsushita, Yoshikazu Uenosono, Takaaki Arigami, Shigehiro Yanagita, Yuka Nishizono, Takahiko Hagihara, Munetsugu Hirata, Naoto Haraguchi, Hideo Arima, Yuko Kijima, Hiroshi Kurahara, Kosei Maemura, Hiroshi Okumura, Sumiya Ishigami, Shoji Natsugoe

**Affiliations:** Department of Digestive Surgery, and Breast and Thyroid Surgery, Graduate School of Medicine, Kagoshima University, Kagoshima, Japan

## Abstract

**Background:**

Esophageal squamous cell carcinoma is an aggressive gastrointestinal tract cancer. To date, the presence of circulating tumor cells (CTC) has been reported as a prognostic factor in peripheral blood from patients with gastrointestinal cancers.

**Methods:**

The CellSearch system was used to isolate and enumerate CTCs. A total of 90 patients with esophageal squamous cell carcinoma who received chemotherapy or chemoradiotherapy were enrolled. Peripheral blood specimens were collected before and after treatments.

**Results:**

At baseline analysis, CTCs were detected in 25 patients (27.8 %). Overall survival was significantly shorter in patients with than without CTCs. Follow-up blood specimens were obtained from 71 patients. Partial response, stable disease, and progressive disease after treatment were seen in 32, 12, and 27 patients, respectively. CTC positivity after treatment in the progressive disease group (40.7 %) was significantly higher than that of the partial response group (6.3 %). Patients with a change in CTC status from positive to negative had a good prognosis as well as patients without baseline CTCs.

**Conclusions:**

Evaluation of CTCs may be a promising indicator for predicting tumor prognosis and the clinical efficacy of chemotherapy or chemoradiation therapy in patients with esophageal squamous cell carcinoma.

**Electronic supplementary material:**

The online version of this article (doi:10.1245/s10434-015-4392-8) contains supplementary material, which is available to authorized users.

Esophageal squamous cell carcinoma (ESCC) is an aggressive gastrointestinal tract cancer.^1^ Even after curative surgical resection, some patients develop recurrent disease. To date, the presence of circulating tumor cells (CTCs) in blood specimens obtained from patients with gastrointestinal tract cancers, including ESCC, has been reported.^2^–^5^ Early detection of CTCs may be important information prior to various treatments, including surgery, chemotherapy, and chemoradiation therapy (CRT). For instance, if CTCs in peripheral blood are confirmed before surgery, the induction of neoadjuvant therapy may be indicated. Accordingly, the presence or absence of CTCs may impact the timing of surgical intervention. Furthermore, the presence of CTCs would be a useful indicator to evaluate the clinical effects of chemotherapy and CRT. Until now, the detection of rare CTCs has been attempted by a molecular biological approach, such as reverse-transcription polymerase chain reaction (RT-PCR) and flow cytometry, in gastrointestinal tract cancers,^5^–^11^ although the clinical significance of CTCs in ESCC remains unclear. In particular, RT-PCR has been reported to be a useful tool to detect CTCs and predict tumor progression in ESCC patients.^12^ However, high sensitivity and reproducibility would be required for the detection of CTCs from blood specimens with the molecular approach. The CellSearch System (Janssen Diagnostics, LLC, Raritan, NJ) has been developed to identify CTCs in blood, and it has clinical utility to predict tumor progression and prognosis in patients with metastatic breast and prostate cancers.^13^,^14^ There are a few reports regarding CTCs using the CellSearch System in ESCC patients.^15^ Our hypothesis was that CTCs in ESCC patients are associated with progression and prognosis. In this study, CTCs were evaluated using the CellSearch system in ESCC patients, and their clinical impact was assessed.

## Materials and Methods

### ESCC Cell Line

To evaluate the sensitivity of the CellSearch system in vitro, five esophageal squamous cell carcinoma cell lines, TE8, TE9 (Riken BioResource Center), KYSE50, KYSE220, and KYSE270, were used.^16^ KYSE50 was cultured in DMEN (Miltenyi Biotec K.K., Tokyo, Japan). KYSE220, KYSE270, TE8, and TE9 were cultured in a medium with a 1 to 1 mixture of RPMI 1640 (Nissui Pharmaceutical Co., Ltd., Tokyo, Japan) and F-12 HAM (Sigma-Aldrich Co. LLC., USA). Each culture medium was supplemented with 10 % fetal calf serum (Mitsubishi Kasei, Tokyo, Japan), 100 units/mL of penicillin, and 100 units/mL of streptomycin. Cancer cells were grown at 37 °C in a humidified atmosphere containing 5 % CO_2_, as previously described.^16^,^17^

### Clinical Study Design

ESCC patients who were treated at Kagoshima University Hospital were analyzed using prospectively collected data. Informed consent was obtained from all patients in accordance with the ethical standards of the Committee on Human Experimentation of Kagoshima University Hospital, Japan.

A total of 103 consecutive patients with advanced ESCC were enrolled from July 2011 to January 2014. Thirteen patients with another advanced cancer, such as gastric, colorectal, or prostate cancer, were excluded from this study. A total of 90 patients without previous treatment were enrolled for the analysis. Fifty-seven patients (63.3 %) were still alive at the time of the analysis. Patients were classified and staged on the basis of the criteria in the seventh edition of the tumor-node-metastasis (TNM) classification of esophageal cancer established by the Union for International Cancer Control (UICC).^18^ The clinicopathological features are shown in Table 1. Distant metastases were observed in 28 patients. Twenty patients had hematogenous metastases. Pleural dissemination was found in six patients, and distant lymph node metastases involving, for example, the para-aortic, axial, and inguinal lymph nodes, were identified in ten patients. The number of patients in each clinical stage was 2 cases in II, 12 cases in IIIa, 12 cases in IIIb, 36 cases in IIIc, and 28 cases in IV, respectively.

**Table 1:** Characteristics of patients with ESCC (*n* = 90)

		Total (*n* = 90)
Gender	Male/female	78/12
Age	Average (range)	65.0 years (46–98)
cT factor	1/2/3/4a/4b	4/4/49/4/29
cN factor	0/1/2/3	1/12/28/49
cM factor	0/1	62/28
cStage	IIa/IIIa/IIIb/IIIc/IV	2/12/12/36/28
Serum CEA	Negative/positive	75/15
Serum SCC	Negative/positive	33/57
CRP	<0.4/≥0.4	39/51
Treatment	Chemotherapy/CRT	26/64
Surgical resection after treatment	No/yes	74/16

*cT cN cM factors* were diagnosed using UICC 7th edition

*cT factor* depth of primary tumor, *cN factor* lymph node metastatic status, *cM factor* status of distant metastasis, including distant lymph node metastasis, *CEA* carcinoembryonic antigen, *SCC* squamous cell carcinoma antigen, *CRP* C-reaction protein, *CRT* chemoradiation therapy

All patients were tested with serum tumor markers, such as carcinoembryonic antigen (CEA), squamous cell carcinoma antigen (SCC), and C-reactive protein (CRP), as an inflammatory marker. Sixty-four patients received CRT, and 26 patients were treated with chemotherapy alone. Chemotherapy was provided by a high-dose FP regimen using 5-fluorouracil and cisplatin or by a DCS regimen using docetaxel, cisplatin, and S-1. CRT consisted of intravenous chemotherapy using an FP regimen and a total radiation dose of 40–60 Gy in the same period.^19^,^20^ In this study, 36 cases were treated as a neoadjuvant therapy, and another 54 patients were treated as an unresectable case. A total of 16 patients underwent surgical resection after treatment. Fourteen patients received surgical resection after neoadjuvant therapy, and two cases who were diagnosed with progressive disease after chemotherapy received surgical resection as a salvage surgery.

Peripheral blood was collected for the baseline analysis before starting treatments, and peripheral blood specimens were obtained from 15 healthy volunteers without cancers as a control group after obtaining their consent.

### Isolation and Detection of Circulating Tumor Cells Using the CellSearch System

The 10-mL blood specimens were drawn into the CellSave Preservative Tubes (Janssen Diagnostics, LLC). Specimens were maintained at room temperature and processed within 72 h after collection. All assessments were performed by technical assistants who were blinded to the patients’ clinicopathological data. The CellSearch system was used for the isolation and enumeration of CTCs, and 7.5 mL of the 10 mL in the tubes were assessed by this assay. It consists of a semiautomated system for preparation of a sample and is used with the CellSearch Epithelial Cell Kit. The procedure enriches the sample for cells expressing EpCAM with antibody-coated magnetic beads, and it labels the nucleus with the fluorescent nucleic acid dye 4, 6-diamidino-2-phenylidole dihydrochloride (DAPI). Fluorescently labeled monoclonal antibodies specific for leukocytes (CD45-allophycocyan) and epithelial cells (cytokeratin 8, 19, 19-phycoerythin) are used to distinguish epithelial cells from leukocytes. The identification and enumeration of CTCs were performed with the use of the Celltracks analyzer II, a semiautomated, fluorescence-based, microscopy system that permits computer-generated reconstruction of cellular images. CTCs were defined as nucleated cells lacking CD45 and expressing cytokeratin (Supplement Fig. 1A). The criteria used in the CellSearch system to define a tumor cell have been described previously.^13^,^14^ Results are expressed as the number of CTCs per 7.5 mL of whole blood.

### Cell-Spiking Experiments for Sensitivity and Linearity of the CellSearch System

A cell-spiking study was done to investigate the sensitivity and linearity of the CellSearch system by spiking a series of serial dilutions of TE8, TE9, KYSE50, KYSE220 and KYSE270 (1000, 100, 50, 10, 5, and 0 cells) into whole blood obtained from a normal healthy volunteer without cancer. This in vitro experiment was repeated three times to validate its reproducibility.

### Clinical Follow-up

All patients were followed-up by physical examinations and routine blood tests including serum tumor marker tests (CEA and SCC) every month and computed tomography (CT) examination every 3 months. Follow-up data were obtained with a median follow-up period of 10.3 (range 0.3–36.4) months.

### Statistical Analysis

Chi square and Fisher’s exact tests were used to compare CTC status with categorical clinicopathological factors. The Kaplan–Meier method was used for survival analysis, and the differences in survival were examined by the log-rank test. Prognostic factors were assessed by univariate and multivariate analyses (Cox proportional hazard regression model). All statistical calculations were performed using SAS statistical software (SAS Institute. Inc., Cary, NC). *p* < *0.05* was considered significant.

## Results

### Sensitivity of the CellSearch System in the Cell-Spiking Study

Regression analysis of the number of observed tumor cells versus the number of expected tumor cells produced a correlation coefficient of 0.980 (Supplement Fig. 1B).

### CTC Analysis in Healthy Volunteers

No CTCs were identified in all blood specimens of the 15 healthy volunteers. In this study, a positive result was defined as the presence of one or more CTCs per 7.5 mL of blood.

### Analysis of CTCs in Clinical Blood Samples of Patients with ESCC

CTCs were identified in 25 of 90 patients (27.8 %) before treatment. The CellSearch system demonstrated 12 patients with one CTC, 4 patients with two CTCs, 5 patients with 3–9 CTCs, 2 patients with 10–99 CTCs, and 2 patients with ≥100 CTCs (Supplement Fig. 2). CTCs were identified in 7 patients (19.4 %) in the neoadjuvant therapy group and in 18 patients (33.3 %) in the unresectable group.

### Relationship Between CTC Status and Clinicopathological Findings

CTCs were found in 25 % with cT1-2 tumor, 32.7 % with cT3 tumor, and 21.2 % with cT4 tumor. Positive rates of CTC by clinical lymph node status were 0.0 % in cN0, 16.7 % in cN1, 28.6 and 30.6 % in cN3. There was no relationship between CTC status and tumor invasion or lymph node metastasis. CTCs were found in 17.7 % without distant metastases and 50.0 % with distant metastases. Finally, CTCs were observed 26.9 % patients in stage II-IIIb, 11.1 % in stage IIIc, and 50.0 % in stage IV. The positive rate of CTCs was significantly related with distant metastasis (*p* = 0.002) and with clinical stage status (*p* = 0.002; Table 2). Furthermore, the CTC detection rate was 100 % in pleural dissemination, 50.0 % in hematogenous metastasis, and 40.0 % in distant lymph node metastasis. There were significant relationships between CTC status and pleural dissemination or hematogenous metastases (*p* < 0.0001, *p* = 0.015; Supplementary Table 1).

**Table 2:** Clinical characteristics and CTC status (*n* = 90)

	CTC positive (*n* = 25)	CTC negative (*n* = 65)	*p* value
Gender			
Male	22	56	0.816
Female	3	9	
Age			
<65	14	33	0.656
≥65	11	32	
cT factor			
1–3	18	39	0.284
4	7	26	
cN factor			
0–2	10	30	0.598
3	15	35	
cM factor			
0	11	51	0.002
1	14	14	
c Stage			
II–IIIb	7	19	0.002
IIIc	4	32	
IV	14	14	
Serum CEA			
Negative	18	57	0.085
Positive	7	8	
Serum SCC			
Negative	11	22	0.374
Positive	14	43	
CRP			
<0.4	9	30	0.381
≥0.4	16	35	
Treatment			
Chemotherapy	13	13	0.004
CRT	12	52	
Surgical resection			
No	22	52	0.359
Yes	3	13	

Patients receiving chemotherapy had significantly higher CTC positivity than those receiving CRT (*p* = 0.004). Serum CEA, serum SCC, and CRP showed no relationship with CTCs.

**Fig. 1:**
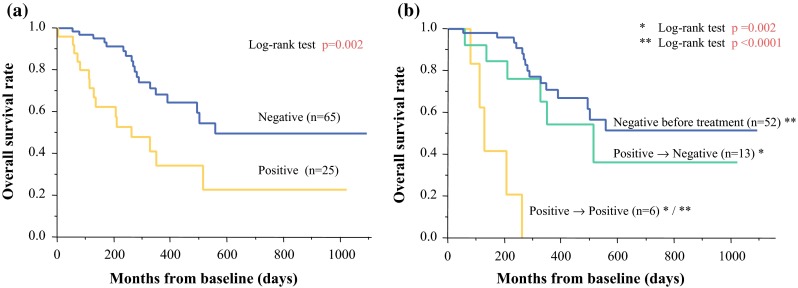
**a** Twenty-five of 90 patients had positive CTCs. The overall survival rate was significantly lower in patients with CTCs than in those without CTCs (*p* = 0.002). The median survival times were 261 days in patients with CTCs and 557 days in patients without CTCs. b In 71 cases, blood samples were collected before and after treatment. Patients with CTCs before and after treatment showed a poorer prognosis than patients without CTCs before and after treatment (*p* < 0.0001). Patients who became CTC-negative after treatment had a good prognosis, similar to that of patients without CTCs before treatment (*p* = 0.002)

The overall survival rate was significantly lower in patients with CTCs than in those without CTCs (*p* = 0.002; Fig. 1a). The median survival time (MST) of CTC-positive patients was 261 days and that of CTC-negative patients was 557 days.

### Univariate and Multivariate Analyses of the Detection of CTCs and Survival

Univariate analyses demonstrated that CTC positivity (*p* = 0.003), multiple lymph node metastases (*p* = 0.002), distant metastases (*p* < 0.0001), serum CRP (*p* = 0.003), chemotherapy (*p* = 0.002), and surgical resection (*p* = 0.002) were significantly related to overall survival (Table 3). Multivariate regression analysis showed that CTC and serum CRP were independent prognostic factors (*p* = 0.021 and *p* = 0.034).

**Table 3:** Univariate and multivariate analyses (*n* = 90)

Independent factors	Univariate analysis		Multivariate analysis	
	Hazard ratio (95 % CI)	*p* value	Hazard ratio (95 % CI)	*p* value
Age (<65 vs. ≥5)	0.89 (0.44–1.76)	0.728		
Gender (female vs. male)	1.87 (0.79–3.97)	0.146		
CTC before treatment (*p* vs. *n*)	2.91 (1.44–5.80)	0.003	2.56 (1.15–5.68)	0.021
cT factor (cT4 vs. cT1-3)	1.70 (0.83–3.38)	0.144		
cN factor (cN3 vs. cN0-2)	3.31 (1.55–7.88)	0.002	2.26 (0.89–6.24)	0.089
cM factor (cM1 vs. cM0)	5.68 (2.72–12.06)	<0.0001	2.18 (0.82–5.81)	0.117
CEA (positive vs. negative)	1.18 (0.44–2.68)	0.714		
SCC (positive vs. negative)	1.35 (0.67–2.86)	0.412		
CRP (≥0.4 vs. <0.4)	3.01 (1.44–6.87)	0.003	2.33 (1.06–5.56)	0.034
Treatment (chemotherapy vs. CRT)	3.30 (1.60–6.71)	0.002	1.38 (0.54–3.43)	0.499
Surgical resection (yes/no)	0.210 (0.05–0.60)	0.002	0.45 (0.10–1.52)	0.211

*CI* confidence interval

### Evaluation of Therapeutic Efficacy by CTC Status Before and After Treatment

Blood specimens for second-line analysis were obtained from 71 patients. The average interval for CTC examination was 77.9 (range 35–201) days, and that for clinical diagnosis of treatment efficacy was 98.9 (range 35–210) days. Partial response (PR), stable disease (SD), and progressive disease (PD) were seen in 32, 12, and 27 cases, respectively. CTC positivity after treatment was 21.1 % for all cases, 40.7 % in the PD group, 16.7 % in the SD group, and 6.3 % in the PR group. At the CTC analysis after treatment, patients with CTC showed significantly poor prognosis rather than patients without CTC (*p* = 0.005; Supplement Fig. 3). Six patients had positive CTCs before and after treatment, 13 patients changed from CTC-positive to negative, 9 patients changed from CTC-negative to positive, and 43 patients remained CTC-negative. Six of 9 cases (66.7 %) whose CTC changed from negative to positive showed PD, and 8 of 13 cases (61.5 %) whose CTC changed from positive to negative showed PR. Therefore, CTC status correlated with therapeutic efficacy (*p* = 0.034), and CTC positivity after treatment was significantly higher in the PD group than in the PR group (*p* = 0.004; Table 4). The MST was 128 days in patients who remained CTC-positive, 514 days in patients who changed from CTC-positive to negative, and more than 557 days in patients without CTCs before treatment. Patients with CTCs before and after treatment showed significantly poorer prognosis than patients whose CTC changed positive to negative (*p* = 0.002) and patients without CTCs before treatment (*p* < 0.0001). In contrast, patients whose CTC changed positive to negative had a good prognosis, as well as patients without CTCs before treatment (*p* = 0.323; Fig. 1b).

**Table 4:** Evaluation of therapeutic efficacy by CTC status change (*n* = 71)

Treatment efficacy	CTC status change					
	Positive to positive	Negative to positive	Positive to negative	Negative to negative	Total	Second-line CTC positivity
PR	0	2	8	22	32	6.3 % (2/32)
SD	1	1	2	8	12	16.7 % (2/12)
PD	5	6	3	13	27	40.7 % (11/27)
Total	6	9	13	43	71	21.1 % (15/71)
					*p* = 0.034	*p* = 0.004

*PR* partial response, *SD* stable disease, *PD* progressive disease

## Discussion

Various methods for detection of CTCs have been attempted by molecular biological approaches, such as RT-PCR assays in gastrointestinal tract cancer.^5^–^11^ Several investigators have reported that the detection of CTCs using RT-PCR is useful to predict the prognosis in patients with ESCC.^6^,^12^ However, the clinical significance of CTCs remains unclear, because molecular techniques by mRNA amplification may detect not only live tumor cells but also dead tumor cells.

In this study, a morphological technique was used for CTC detection, and this method can identify viable cancer cells within peripheral blood. The CellSearch^®^ System (CSS) began in 1999 and led to the first standardized, FDA-cleared, semiautomatic system that can capture and quantify circulating tumor cells in peripheral blood. To date, many studies using CSS have been published in patients with breast, prostate, colorectal, and other solid cancers, and these reports indicated the clinical usefulness of CSS monitoring as a “liquid biopsy” for patients with metastatic cancers.^13^–^15^,^21^ However, clinical studies of a large number of ESCC patients have not been reported. In the present study, we evaluated the relationship between CTC and clinicopathological factors or prognosis. The presence of CTCs was significantly correlated with distant metastases, such as pleural dissemination and hematogenous metastases (*p* = 0.002), although positive expression of CTCs in distant metastases was lower than expected. For CTC-positive rates, sensitivity is higher with RT-PCR than with CSS. However, there might be false-positives, because the PT-PCR method is not able to demonstrate cancer cells visually. On the other hand, the false-positive rate with the CSS is thought to be extremely low, because it is possible to morphologically confirm the presence of cells. Some cases, however, might be missed (false-negatives), because the EpCAM as an epithelial marker is not always expressed in all CTCs, and these EpCAM-negative CTCs may be undetectable by the CSS. One of the reasons for this is the presence of an epithelial-mesenchymal transition (EMT) in CTCs. The EMT is one of the important pathways for tumor cells to dissociate and migrate into the peripheral blood stream.^22^ Yu reported that CTCs simultaneously expressed mesenchymal and epithelial markers, but mesenchymal cells were highly enriched in CTCs.^23^ EpCAM-positive CTCs may be the tip of the iceberg of total CTCs. Although CSS is able to detect only epithelial cancer cells, EpCAM-CTC was associated with a worse prognosis in patients with ESCC than in CTC-negative cases (MST: 261 vs. 557 days, *p* = 0.002) in the present study. This result obviously indicated the clinical significance of EpCAM-CTC as a prognostic indicator.

Some reports discussed the relationships between the CTC transition and prognosis.^24^–^26^ Smerage et al. demonstrated that metastatic breast cancer patients with persistently increased CTCs after one cycle of first-line chemotherapy showed a poorer prognosis than patients without CTCs.^24^ In the present study, CTC expression at the second-line assessment and therapeutic efficacy were highly related (Table 4; *p* = 0.034). Patients with CTC after treatment showed a significantly poor prognosis than patients without CTC (*p* = 0.005; Supplement Fig. 3). In addition, the prognosis of patients whose CTC changed positive to negative was improved as well as that of patients without CTCs before treatment (Fig. 1b). Furthermore, patients with CTC after treatment showed a shorter progression-free survival time rather than patients without CTC (mean time; 73 vs. 326 days, *p* < 0.001). Treatment efficacy was assessed about 3 weeks after second-line samples were collected. Therefore, the CTC examination as a “liquid biopsy” may be able to predict therapeutic efficacy earlier than conventional imaging examinations.

## Conclusions

The evaluation of CTCs in peripheral blood is a useful tool for predicting tumor progression and prognosis in patients with ESCC. Furthermore, the monitoring of CTC status may serve as a promising approach for calculating on the therapeutic efficacy.

## Electronic supplementary material

Below is the link to the electronic supplementary material.

Supplement Fig. 1 A Circulating tumor cells (CTCs) are isolated by immunomagnetic beads coated with antibodies against the epithelial cell adhesion molecule (EpCAM) and are identified by cytokeratin (CK) positivity, positive nuclear staining (DAPI), and CD45 negativity. The figure shows a cell line (KYSE220: moderately differentiated SCC) as a positive control. B The spiking study of the number of observed tumor cells versus the number of expected tumor cells shows a correlation coefficient (*R* ^2^) of 0.980. A series of serial dilutions of TE8, TE9, KYSE220, and KYSE270 (1000, 100, 50, 10, 5, and 0 cells) into whole blood from a normal healthy volunteer who did not have any cancer was assessed by the CellSearch system. Supplement Fig. 2 CTCs are detected in 25 patients (27.8 %). The range of CTC counts was 1 to >25,000. CTCs were not found in any samples from healthy volunteers. Supplement Fig. 3 In 71 cases, blood samples were collected before and after treatment. Fifteen patients with CTCs after treatment showed a poorer prognosis than patients without CTCs treatment (*p* = 0.005) (PPTX 123 kb)

Supplementary material 2 (DOC 34 kb)
