# Supplementary material for: Clinical Significance of Circulating Tumor Cells in Peripheral Blood of Patients with Esophageal Squamous Cell Carcinoma
Source: Ann Surg Oncol. 2015 Feb 5;22(11):3674–80. doi: 10.1245/s10434-015-4392-8 (PMC4565870; doi:10.1245/s10434-015-4392-8)
Supplement: Supplementary file 1 — Supplement Fig. 1 A Circulating tumor cells (CTCs) are isolated by immunomagnetic beads coated with antibodies against the epithelial cell adhesion molecule (EpCAM) and are identified by cytokeratin (CK) positivity, positive nuclear staining (DAPI), and CD45 negativity. The figure shows a cell line (KYSE220: moderately differentiated SCC) as a positive control. B The spiking study of the number of observed tumor cells versus the number of expected tumor cells shows a correlation coefficient (R 2) of 0.980. A series of serial dilutions of TE8, TE9, KYSE220, and KYSE270 (1000, 100, 50, 10, 5, and 0 cells) into whole blood from a normal healthy volunteer who did not have any cancer was assessed by the CellSearch system. Supplement Fig. 2 CTCs are detected in 25 patients (27.8 %). The range of CTC counts was 1 to >25,000. CTCs were not found in any samples from healthy volunteers. Supplement Fig. 3 In 71 cases, blood samples were collected before and after treatment. Fifteen patients with CTCs after treatment showed a poorer prognosis than patients without CTCs treatment (p = 0.005) (PPTX 123 kb) [file 10434_2015_4392_MOESM1_ESM.pptx]

## Slide 1
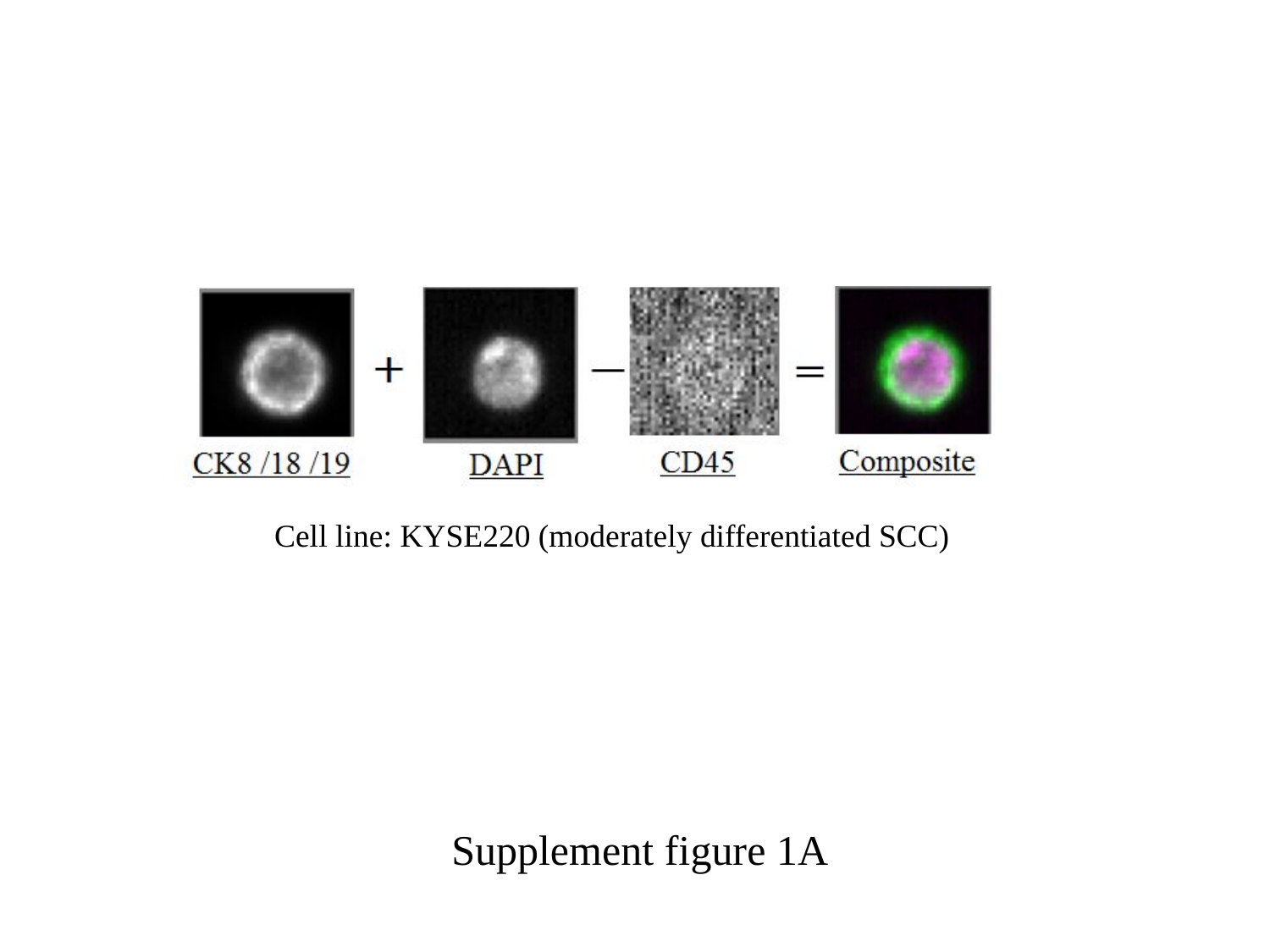

Cell line: KYSE220 (moderately differentiated SCC)
Supplement figure 1A

## Slide 2
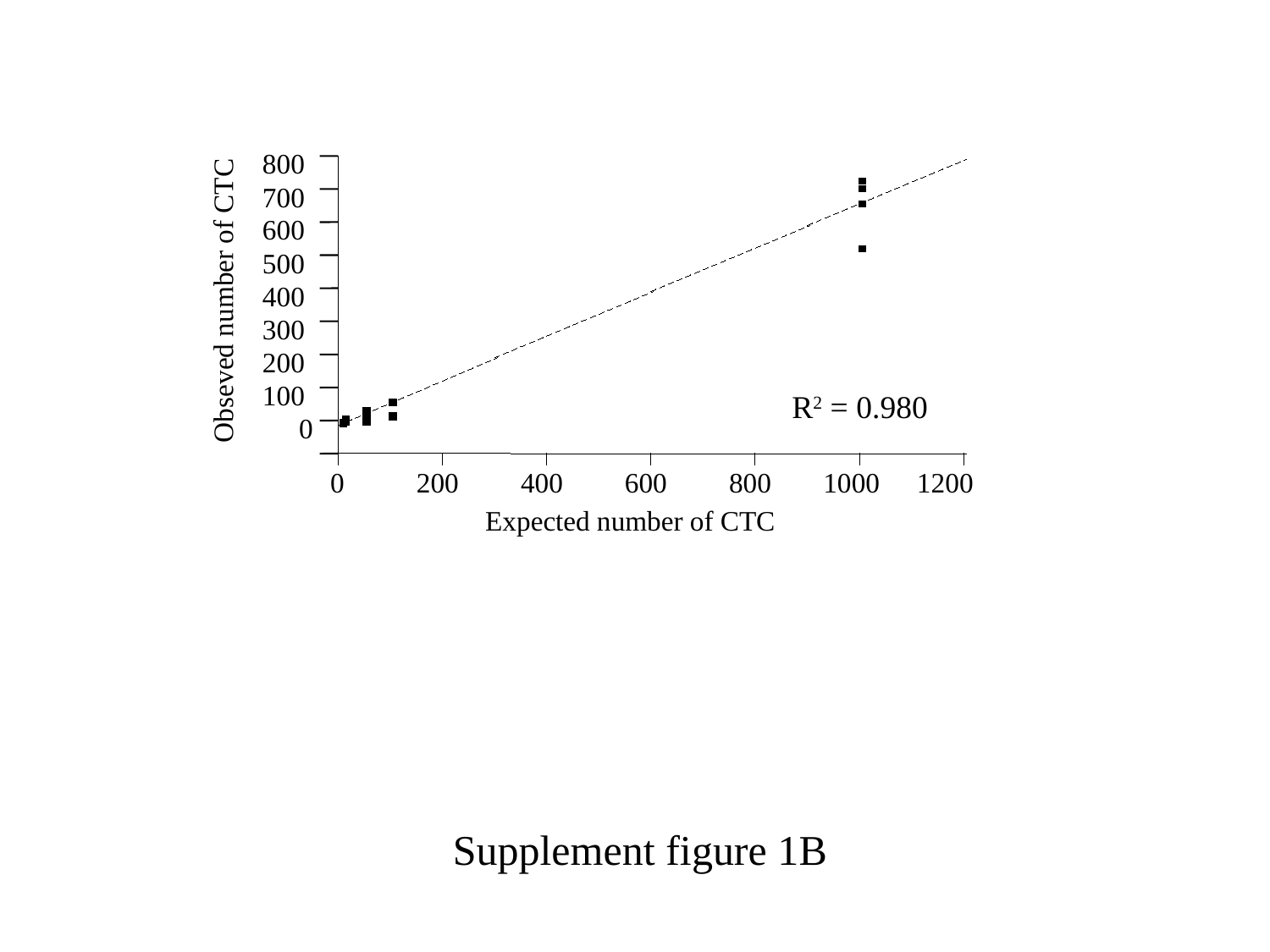

800
700
600
500
400
Obseved number of CTC
300
200
100
0
0
200
400
600
800
1000
1200
Expected number of CTC
R2 = 0.980
Supplement figure 1B

## Slide 3
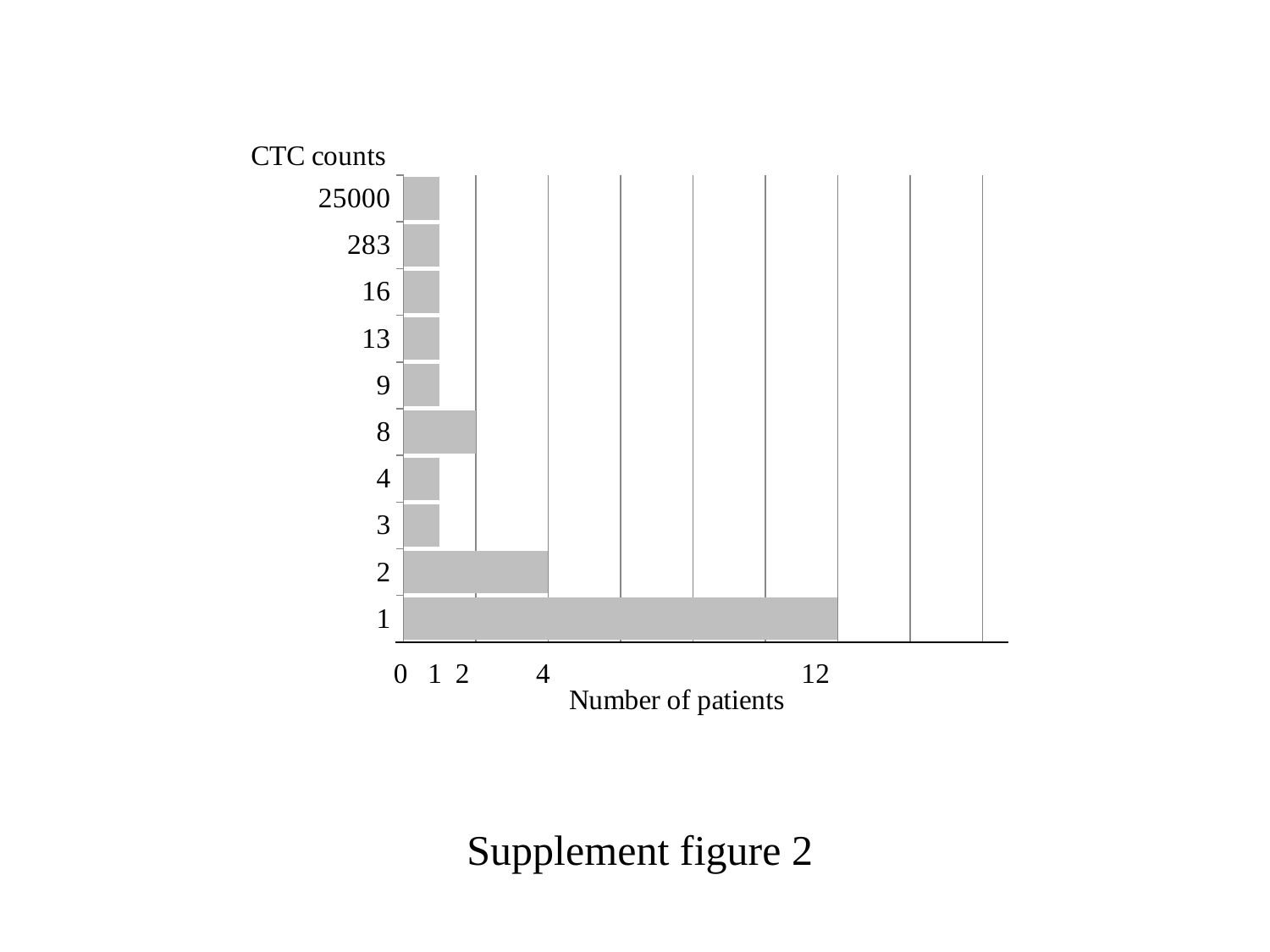

### Chart: CTC counts
| Category | 系列 1 |
|---|---|
| 1 | 12.0 |
| 2 | 4.0 |
| 3 | 1.0 |
| 4 | 1.0 |
| 8 | 2.0 |
| 9 | 1.0 |
| 13 | 1.0 |
| 16 | 1.0 |
| 283 | 1.0 |
| 25000 | 1.0 |Supplement figure 2

## Slide 4
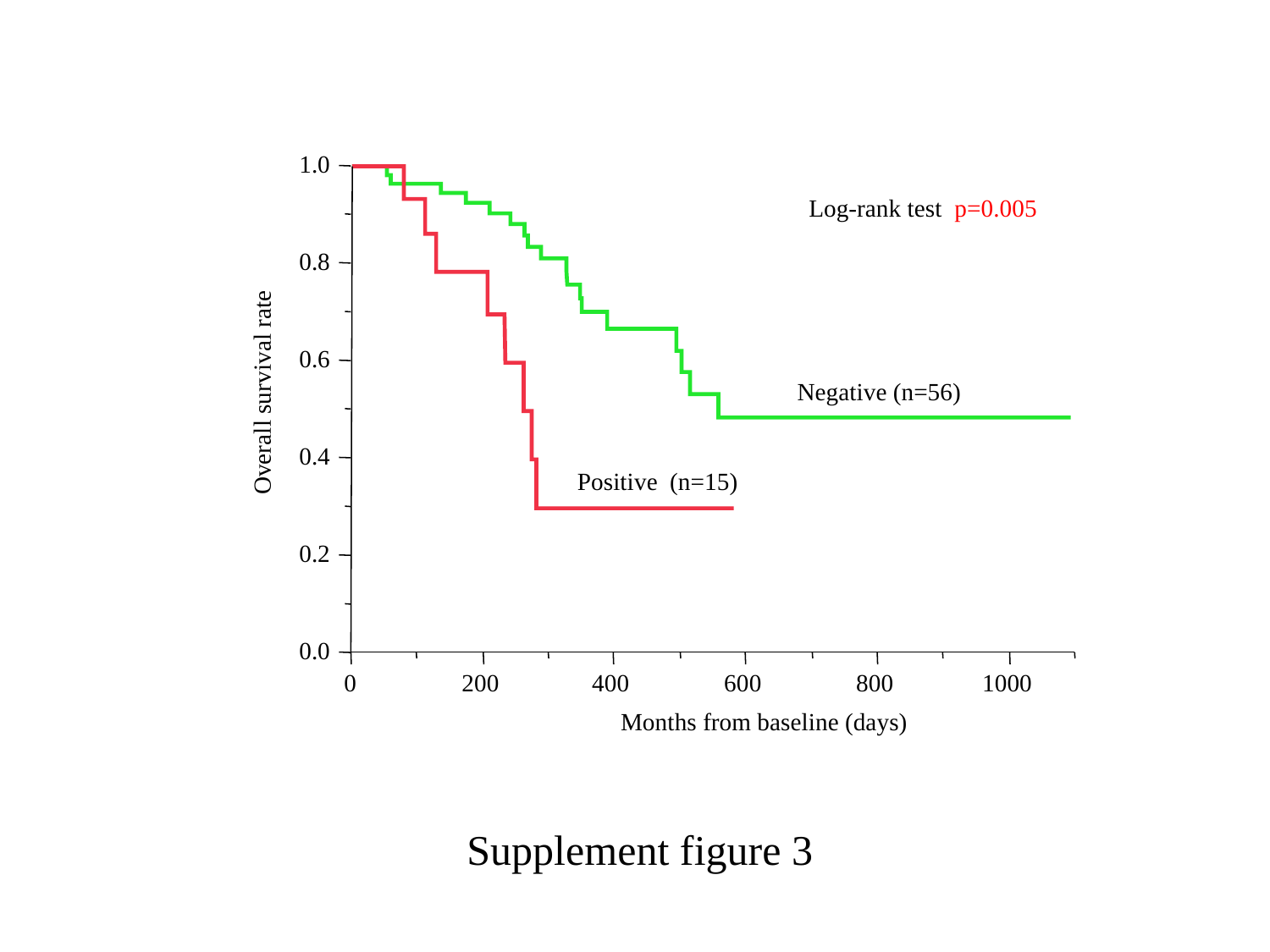

1.0
0.8
0.6
0.4
0.2
0.0
0
200
400
600
800
1000
Overall survival rate
Months from baseline (days)
Log-rank test p=0.005
 Negative (n=56)
 Positive (n=15)
Supplement figure 3
