# Supplementary material for: Clinical Significance of Circulating Tumor Cells in Peripheral Blood of Patients with Esophageal Squamous Cell Carcinoma
Source: Ann Surg Oncol. 2015 Feb 5;22(11):3674–80. doi: 10.1245/s10434-015-4392-8 (PMC4565870; doi:10.1245/s10434-015-4392-8)
Supplement: Supplementary file 2 — Supplementary material 2 (DOC 34 kb) [file 10434_2015_4392_MOESM2_ESM.doc]

**Supplementary table 1.** CTC positivity with unresectable factors.

| **Distant metastasis** | **CTC positivity** | | |  |
| --- | --- | --- | --- | --- |
|  | **50.0%** |  | **(14/28)** | ***p = 0.002*** |
| **Pleural dissemination** | **100%** |  | **（6/6）** | ***p < 0.0001*** |
| **Hematogenous metastasis** | **50.0%** |  | **(10/20)** | ***p = 0.015*** |
| Liver | 50.0% |  | （5/10） | *p=0.113* |
| Lung | 55.6% |  | （5/9） | *p=0.064* |
| Bone | 100% |  | （6/6） | *p<0.0001* |
| Thyroid gland | 0.0% |  | （0/2） | *p=0.251* |
| Adrenal gland | 100% |  | (1/1) | *p=0.108* |
| Stomach (intramural metastasis) | 0.0% |  | （0/2） | *p=0.251* |
| **Distant lymph node metastasis** | **40.0%** |  | **（4/10）** | ***p = 0.384*** |
